# Correlation of subfoveal choroidal thickness with axial length, refractive error, and age in adult highly myopic eyes

**DOI:** 10.1186/s12886-018-0791-5

**Published:** 2018-05-29

**Authors:** Bingqian Liu, Yan Wang, Tao Li, Ying Lin, Wei Ma, Xiaohong Chen, Cancan Lyu, Yonghao Li, Lin Lu

**Affiliations:** 1 State Key Laboratory of Ophthalmology, Zhongshan Ophthalmic Center, Sun Yat-sen University, 54 South Xianlie Road, Guangzhou, 510060 Guangdong China; 2 Department of Ophthalmology, Shenzhen Hospital of Southern Medical University, Shenzhen, China

**Keywords:** High myopia, Subfoveal choroidal thickness, Axial length, Age, Refractive error, Correlation analysis

## Abstract

**Background:**

Subfoveal choroidal thickness (SFCT) in highly myopic eyes was found to be correlated with increasing age, refractive error (spherical equivalent), and axial length. Which factor is the most significant predictor of SFCT remains controversial.

**Methods:**

A hospital-based cohort of highly myopic eyes (with spherical equivalent equal to or over 6.00 diopter) were retrospectively screened. Data from only right eye in those bilateral high myopia, and unilateral high myopia in any eye, were used for analysis. Correlations among the four biometric factors were analyzed. Linear correlation was performed to analyze the predictors of SFCT.

**Results:**

A cohort of 312 eyes from 312 adults (98 men) was enrolled. Statistical analysis showed that axial length (*R* = − 0.592), spherical equivalent (*R* = − 0.471), and age (*R* = − 0.296) were significantly correlated with SFCT (*P* < 0.001). No significant correlation was found between age and axial length, or age and spherical equivalent. Partial correlation with controlled age confirmed that axial length (*R* = − 0.628) was a more significant predictor of SFCT than spherical equivalent (*R* = − 0.507).

**Conclusions:**

SFCT was inversely correlated with increasing age, spherical equivalent and axial length, with axial length as the most significant predictor of SFCT, in adult highly myopic eyes.

## Background

Myopia is a significant public health concern worldwide [1]. The prevalence of myopia in teenagers and young adults has reached up to 90% in East Asia, up to 50% in United States and Europe, and the overall prevalence of myopia is still increasing [1–11]. High myopia, featured by progressive elongation of the eyeball, is associated with potentially blinding complications such as retinal detachment, macular schisis, macular hole, chorioretinal atrophy, choroidal neovascularization, and peripapillary cavity [7, 8, 12–15]. It is estimated that 1–2% of the population in western countries exhibits high myopia, with a much higher prevalence in East Asian. Population-based studies showed high myopia to be the 1st to 3rd most frequent cause of blindness, and the prevalence of visual impairment attributable to high myopia ranged from 0.1 to 0.5% in European studies and from 0.2 to 1.4% in Asian studies [16].

The severity of myopic macular degeneration was found strongly associated with subfoveal choroidal thicknesses (SFCT) in a recent study [17]. Accurate measurement of choroidal thickness may provide some information about myopic pathologic conditions. The development of enhanced depth imaging (EDI) of spectral-domain optical coherence tomography (SD-OCT) allows for the evaluation of choroidal thickness in vivo [18–20].

Choroidal thickness in highly myopic eyes was reported to be correlated with increasing age, spherical equivalent, and axial length, and SFCT could be considered as a useful predictor of visual function [21–25], but which factor is the most significant predictor remains controversial in previous studies, and the data from different population might be variable according to literatures [21, 25]. The goal of this study was to estimate the correlation of SFCT with age, axial length and spherical equivalent in a hospital-based cohort of highly myopic eyes, and to find which factor was the most significant predictor of SFCT.

## Methods

This is a retrospective cross-sectional study, by re-analysis of the project data of “high myopia cohort study in a tertiary eye center”. An informed consent was obtained from all of the included subjects for the project but not specifically for this study. Adult patients (age ≥ 18 years) with high myopia from December 2013 to April 2016 collected by high myopia research group at Fundus Disease Center of Zhongshan Ophthalmic Center were screened. The study followed the tenets of the Declaration of Helsinki and was approved by the Zhongshan Ophthalmic Center Institutional Review Board.

Inclusion criteria was spherical equivalent equal to or over 6.00 diopter (D); Exclusion criteria included poor OCT image quality and those choroid borderlines could not be clearly visualized; history of vitrectomy, sclera buckling, glaucoma, or retinal detachment. Data from only right eye were used for analysis in those with bilateral high myopia. Unilateral high myopia in any eye was also included.

### Measurements

All the participants underwent ophthalmic examinations including assessment of BCVA, intraocular pressure measurement, slit lamp examination, spherical equivalent, axial length, indirect dilated fundus ophthalmoscopy, fundus photography, and OCT for the mentioned project. Axial length was measured using IOL Master (Carl Zeiss Meditec, Jena, Germany). Spherical equivalent was measured by autorefractometry (Canon, Tokyo, Japan). Horizontal and vertical cross hair scans through central fovea were captured by an SD-OCT machine (Heidelberg, Germany) with EDI modality. Choroidal thickness was defined from the outer edge of the hyperreflective line corresponding to the retinal pigment epithelium to the inner surface of sclera [18, 25, 26]. The SFCT was manually measured and averaged by two independent observers from horizontal and vertical scans. Repeatability of SFCT measurements between the scans and observers were analyzed.

### Statistics

Statistical analyses were performed using SPSS 17.0 software. Pearson’s correlation was used to analyze the correlation between any two biometric factors. Analysis of multi-predictors (axial length/spherical equivalent and age) of SFCT were analyzed using stepwise method. Partial correlations with controlled factor were studied. Analysis of linear regression of SFCT with its predictors of axial length, spherical equivalent, and age was performed. *P* < 0.05 was considered statistically significant.

## Results

### Correlations among biometric factors in high myopia

Our cohort comprised 312 eyes from 312 patients (98 men). Table 1 shows the demographic and clinical characteristics. The correlation between the measurements of SFCT performed by two independent observers was highly significant (*r* = 0.96; *p* < 0.001). The correlation between SFCT measurements from vertical scan and horizontal scan was also highly significant (*r* = 0.89, *p* < 0.001).

**Table 1:** Epidemiological data of included eyes

Variable	Minimum	maximum	mean	SD
age (years)	18	88	47.47	14.11
^a^Refractive error (D)	−6.00	−32.00	−14.58	5.52
Axial length (mm)	24.31	36.23	29.45	2.31
SFCT (um)	10.00	337.00	83.77	54.64

*SFCT* subfoveal choroidal thickness

^a^ Eyes underwent refractive or cataract surgery (*n* = 31) were not included

Correlation analysis among the four biometric factors showed that SFCT was significantly negative correlated with axial length (*r* = − 0.592, *p* < 0.001), negative correlated with spherical equivalent (*r* = − 0.471, *p* < 0.001), and negative correlated with age (*r* = − 0.296, *p* < 0.001), respectively, as shown in Table 2. With the increasing of axial length, spherical equivalence and age, the SFCT became gradually thinning. Among these three biometric factors, axial length showed the largest coefficient correlated to SFCT. Axial length and spherical equivalent was highly correlated, as commonly expected (*r* = 0.773, *p* < 0.001). There was no significant correlation between age and axial length. No significant correlation between age and spherical equivalent was found, either.

**Table 2:** Correlations among biometric factors in high myopia

		SFCT	Age	SE
Correlation Coefficients	Age	−0.296		
	SE	− 0.471	0.050	
	AL	−0.592	−0.018	0.773
*P* values (2-tailed)	Age	< 0.001		
	RE	< 0.001	0.1798	
	AL	< 0.001	0.3855	< 0.001

*AL* axial length, *SE* spherical equivalent, *SFCT* subfoveal choroidal thickness

### Predictors of subfoveal choroidal thickness

Given that axial length, spherical equivalent, and age were all correlated significantly with SFCT. Multi-predictors of subfoveal choroidal thickness were analyzed. Adding all the three factors resulted in confounding results using stepwise method. The models of axial length plus age and spherical equivalent plus age were similar in the statistical fitness (Table 3). Partial correlation with controlled age further confirmed that axial length (*R* = − 0.628, *p* < 0.001) was a more significant predictor of SFCT than spherical equivalent (*R* = − 0.507, *p* < 0.001).

**Table 3:** Analysis of multi-predictors (axial length/ spherical equivalent and age) of subfoveal choroidal thickness

				Hypothesis test		95% Confidence Interval for B		Partial correlations
Model		B	SE	t	Sig.	Lower bound	Upper bound	
1	Constant	646.854	34.159	18.936	< 0.001	579.673	714.035	–
	Axial length	−16.804	1.109	−15.153	< 0.001	−18.985	−14.623	−0.628
	Age	−1.422	0.182	−7.832	< 0.001	−1.779	−1.065	−0.385
2	Constant	234.984	12.824	18.324	< 0.001	209.763	260.205	–
	Spherical equivalent	−5.627	0.509	−11.060	< 0.001	−4.626	−6.627	−0.507
	Age	−1.482	0.201	−7.366	< 0.001	−1.878	−1.086	−0.365

Axial length and age, or spherical equivalent and age were predictors for the dependent variable, subfoveal choroidal thickness

### Linear regression of SFCT with predictors

**Fig. 1:**
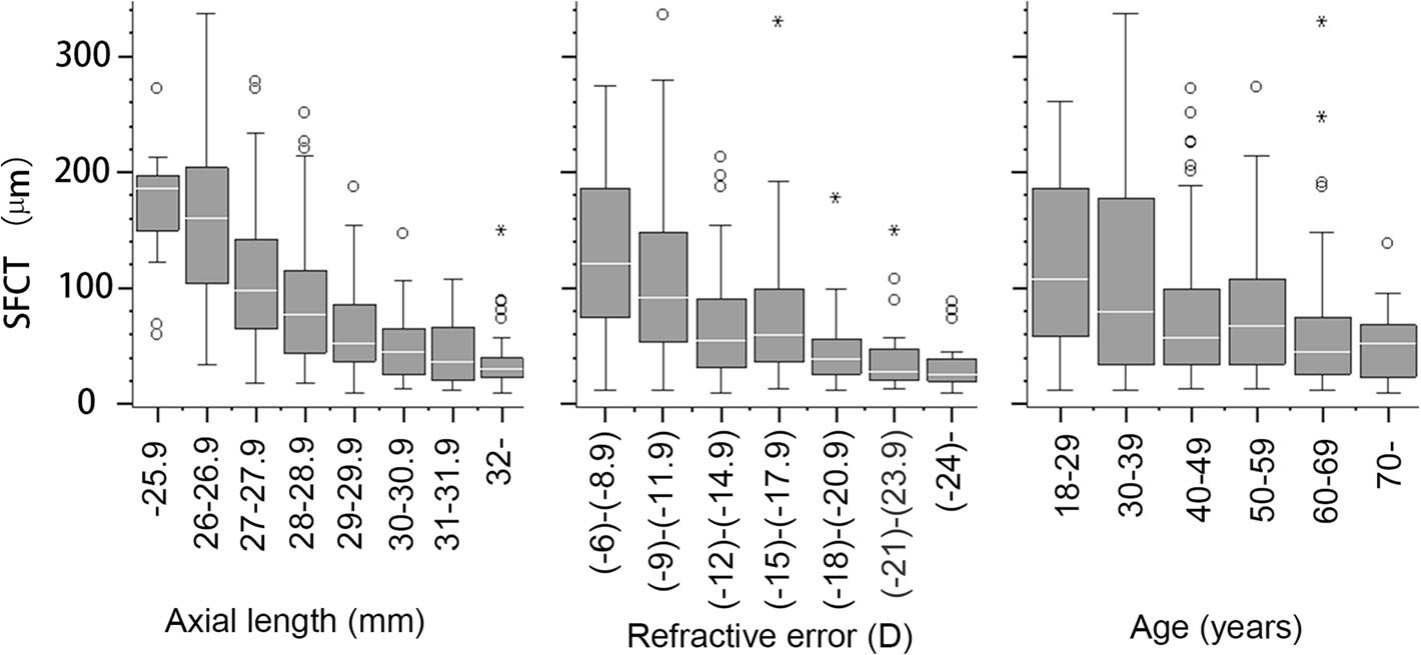
Distribution of SFCT data associated with predictors. Left: SFCT data associated with axial length; Middle: SFCT data associated with refractive error; Right: SFCT data associated with age; о: Outlier values which were between 1.5 and 3 box lengths from either end of the box. *: Extreme values which are more than 3 box lengths from either end of the box

The predictors of SFCT were analyzed using linear regression (Table 4). The distribution of SFCT values in eyes with different levels of predictors was shown in box plot, Fig. 1. The SFCT cohort was grouped by sectioned range of axial length (1 mm), age (1 decade), and spherical equivalent (3D). According to the distribution of data (Fig. 1), the speed of SFCT decrease seemed slowing down with the increasing axial length, age, and spherical equivalent, especially with axial length over 30 mm, age older than 50 years, and spherical equivalent over 15 D.

**Table 4:** Linear regression of SFCT with its predictors of axial length, spherical equivalent, and age

			Hypothesis Test		95% Confidence Interval for B	
Parameters	B	SE	t	Sig.	Lower Bound	Upper Bound
Constant	573.253	35.530	16.134	< 0.001	503.38	643.13
Axial length (mm, *n* = 312)	−16.646	1.200	−13.876	< 0.001	− 19.00	− 14.29
Constant	160.458	8.452	18.985	< 0.001	143.84	177.08
^a^Spherical equivalent (D, *n* = 285)	−5.441	0.545	−9.984	< 0.001	−4.37	−6.51
Constant	148.216	11.755	12.61	< 0.001	125.10	171.34
Age (years, *n* = 312)	−1.372	0.233	−5.89	< 0.001	−1.83	−0.91

*SFCT* subfoveal choroidal thickness (μm), was a dependent variable, *SE* standard error

^a^Eyes underwent refractive or cataract surgery (*n* = 27) were not included

## Discussion

In this study, we found that axial length, rather than spherical equivalent, was the more important predictor of SFCT, different to previous reports from another cohort [21]. In a recent study, by comparing eyes with different degree of myopia and healthy eyes, axial length was found to be determining factor of choroidal thickness in some locations including subfoveal [25]. We suggest that axial length may be more accurate in predicting SFCT in high myopia due to the following reasons: 1) the sample size of our cohort was much larger; 2) the axial length may represent more accurately and directly the extent of choroidal stretch in the posterior pole of highly myopic eyes; 3) the refraction might be confused by development of nuclear cataract, which is very common in adult myopes and could result in further myopic shift; 4) for the eyes with cataract removed, spherical equivalent is not applicable to understanding the myopic status.

The choroidal thickness become gradually thinning with the extension of axial length. However, the SFCT seemed decrease faster in eyes with axial length in the range of 24 to 30 mm compared with those axial length over 30 mm (Fig. 1, left).

The second predictor of SFCT is spherical equivalent. The choroidal thickness become gradually thinning with the increasing of refraction. The speed of SFCT decrease looked much faster in eyes with a spherical equivalent from 6 D to 15 D, compared with those spherical equivalent greater than 15 D (Fig. 1, middle).

Our cohort was from a tertiary eye-care center based population, the myopic degree was probably much higher than other cohorts. The SFCT of our high myopia cohort was 83.77 ± 54.64 μm, much thinner than that in healthy subjects from literatures [27–30]. Our mean SFCT was even thinner than a similar study showing a SFCT of 113.8 ± 53.9 μm in a New York cohort (*n* = 35 eyes), 172.9 ± 72.8 μm in a Japanese cohort (*n* = 110 eyes) [21], 166 ± 88.7 μm in a Spain cohort [22] and 225.87 ± 5.51 μm from a case-control study of young Chinese men in Singapore [31].

Age is associated with SFCT in both high myopic and healthy eyes. In eyes without high myopia, age was found critical for evaluation of SFCT in two healthy Chinese cohorts, one from Guangzhou in southern China, and another from Beijing in northern China [28, 32]. SFCT in subjects older than 60 years of age was much thinner than that in younger subjects, and the SFCT after 60 years of age seemed to keep relatively stable in healthy cohorts [28, 29, 32]. The age of the patients in our cohort was from 18 to 88, but most of the cases were older than 30 years. The speed of SFCT decrease after 50 years of age looked much slower than that from 18 to 49 years of age (Fig. 1, right). Our study did not focus on young myopes. Choroidal thickness in young population [33, 34] might be quite different compared with in older ones.

Our study has several limitations: 1) it is a retrospective cross-sectional study, the data from a hospital-based cohort might represent more severe degree of myopia. 2) some eyes were excluded because of low image quality due to poor focus fixation, or the posterior scleral border was not clear; 3) the correlation of SFCT with other factors, such as gender, visual acuity, retinal thickness, intraocular pressure, ocular biometric parameters, or various maculopathy, was not analyzed, which might be worthy of further investigation; 4) Our cohort does not include patients younger than 18 years. 5) The SFCT may be also affected by other factors including the severity of posterior staphyloma, which was not included in this study.

We included only one eye from each patient to meet statistical requirement. From a recent study, there was larger absolute interocular differences in choroidal thickness of high myopic eyes compared with healthy control eyes [35]. Further study comparing the interocular difference in severity of pathological change and corresponding choroidal thickness and visual function might provide more information for understanding high myopia related changes.

## Conclusion

In summary, SFCT in adult highly myopic eyes was inversely correlated with increasing age, spherical equivalent and axial length. Axial length was a more significant predictor of SFCT than spherical equivalent, or age.

## Abbreviations

D: Diopter
EDI: Enhanced depth imaging
SD-OCT: Spectral-domain optical coherence tomography
SFCT: Subfoveal choroidal thickness
